# The attitudes and knowledge of family physicians regarding malnutrition in the elderly: a call for action

**DOI:** 10.1186/s13584-024-00631-x

**Published:** 2024-09-02

**Authors:** Galia Sheffer-Hilel, Josefa Kachal, Aya Biderman, Danit Rivka Shahar, Shimon Amar

**Affiliations:** 1 Nutrition Sciences Department, Faculty of Sciences at, Tel-Hai Academic College, Kiryat Shmona, Israel; 2 Department of Family Medicine and Siaal Research Center for Family Medicine and Primary Care, Faculty of Health Sciences, Ben-Gurion University of the Negev, Beer-Sheva, Israel; 3 Clalit Health Services, Southern District, Beer-Sheva, Israel; 4 Department of Public Health, The Daniel Abraham International Center for Health and Nutrition, Ben-Gurion University of the Negev, Beer-Sheva, Israel

**Keywords:** Malnutrition, Elderly, Family physician, GLIM

## Abstract

**Background:**

Malnutrition in the elderly places a significant burden on healthcare, social, and aged-care systems, yet it often remains undiagnosed and untreated. This study aims to evaluate family physicians' knowledge and attitudes towards the diagnosis and treatment of malnutrition in the elderly.

**Methods:**

Based on a literature review, an online questionnaire was developed, comprised of seven knowledge-related items and eight attitude-related questions regarding malnutrition in elderly populations. We also assessed the feasibility of including two malnutrition screening questions in regular clinic visits for individuals aged ≥ 70 years.

**Results:**

Surveys were completed by 126 physicians (35% response rate), mean age 47.2 ± 12.6 years; 15.6 ± 12.5 years of practice; 67% females; and 92% board-certified family physicians. Moreover, 77.6% agreed that diagnosing malnutrition is important in patients with decreased appetite. Most respondents demonstrated knowledge of nutritional screening principles (63.5%) and recognized that even obese elderly individuals could be malnourished (83.2%). There was partial agreement (60%) that normal BMI values in the elderly differ from those in younger populations. Almost complete agreement was seen for incorporating two nutritional status questions in medical visits (91%), with physicians expressing willingness to receive training in malnutrition identification and screening tools. Despite challenges such as time constraints and limited knowledge, participants were open to conducting biannual malnutrition risk screening for elderly patients.

**Conclusion:**

We recommend malnutrition screening in primary care followed by malnutrition diagnosis and referral of malnourished patients to the proper intervention.

## Introduction

Malnutrition is a complex condition that has numerous etiologies, that may develop independently or following the interaction between food deprivation and catabolic processes that are linked to disease-related inflammation [1]. In general, it is estimated that 5–26% of older adults are at risk of malnutrition [2]. Older adults, aged 70 years and above, are at even higher risk, due to a range of physiological and social factors, such as frailty, reduced appetite, sensory impairments, poor dentition, swallowing problems, depression, dementia, and even social isolation [3]. Malnutrition could have serious consequences on the individual, such as impaired healing of wounds, reduced immunity [4], higher risk of falls [5], impaired functionality [6], increased mortality risks [7], and decreased quality of life [8]. The burden of malnutrition on health care is significant, taking a toll on resources such as expenses and manpower [9]. According to the recent Vienna Declaration, nutritional care is an emerging human right, in line with the understanding that nutrition is a fundamental aspect of health and well-being. Moreover, it lies at the intersection of the existing human rights to food and to health, i.e., “the right to be fed” [10].

Family Physicians (FP) working in the community serve as the initial point of contact for patients within the healthcare system, therefore, they are ideally positioned to identify patients with malnutrition and refer them to other health care professionals for further assessment and treatment. Although malnutrition in the elderly is a global concern, a fundamental lack of consensus can be seen regarding the criteria for diagnosing malnutrition in clinical settings. The International Classifications of Diseases (ICD) focuses on identifying nutrient deprivation and deficiency [1]. However, the clinical settings and snapshot of global malnutrition has greatly evolved over time, becoming even more complex and intricate. Indeed, this is expected to be addressed in the next ICD to be issued, to provide the clinical nutrition community with more specific coding regarding malnutrition in adults—one that reflects current malnutrition perceptions and may support clinical decision-making in everyday practice [1].

To adequately and accurately diagnose and document malnutrition in medical records, reliable and valid diagnostic tools are needed [11]. One such tool that encompasses the expertise of several nutritional societies is the Global Leadership Initiative on Malnutrition (GLIM)—a global consensus on core criteria for diagnosing malnutrition in adults in all health care settings [12]. The GLIM diagnostic framework is currently being validated for older populations across a variety of settings and clinical conditions [13]. Yet, a recent review of the current literature on identification and treatment of malnutrition in older adults identifies gaps between research evidence clinical practice [14]. This study aimed at examining such gaps in Israel, by assessing FPs’ knowledge and attitudes toward malnutrition diagnosis and treatment in the elderly, with an emphasis on barriers in the use of this diagnostic tool for identifying elderly patients with malnutrition in Israel.

## Methods

### Subjects and research design

In 2022, an online survey was sent to FPs from the four main health maintenance organizations (HMOs) in Israel. To increase the response rate, the questionnaire was kept as short as possible (5–10 min to complete). The electronic questionnaire included seven statements regarding the FPs’ attitudes towards diagnosing and treating malnutrition in the elderly and six statements regarding their knowledge of these topics. For each of the 15 items included in the questionnaire, the respondents were asked to choose their degree of agreement, from the following five options: (1) completely agree; (2) agree; (3) partially agree; (4) disagree; or (5) completely disagree (Appendix 1).

Additional questions were included in the survey regarding barriers and willingness to screen for malnutrition; the respondents were also asked to provide demographic data, including gender, age, years of experience as a physician (including specialization in family medicine), number of years of experience as board certified family physicians, region of practice (north south), type of clinic (urban, rural, or both), and whether there is a dietician at their clinic. A total of about 300 physicians were approached to participate in this study, recruited through three channels: (1) National on-line network of FPs; (2) e-mail sent to all FP's who work in the southern region of the country; and (3) during a large conference for FP's. All data was electronically sent to one data base for analysis. The respondents provided their informed consent prior to completing the questionnaire. The study was approved by the Ethics Committee of. of Ben-Gurion University, Faculty of Health Sciences (06/2022).

### Questionnaire development

Several steps were taken to guarantee the study’s credibility. The research team included dietitians, and Family Physicians which provided diverse perspectives and insight. The focus of the questionnaire is nutritional screening, diagnosing and treating malnutrition. A review of the literature was conducted to provide initial direction for creating the survey, with the included items and questions being related to the various topics that emerged from this review. Moreover, an emphasis was placed on two of the most consistent risk factors for malnutrition: involuntary weight loss and decreased appetite; In addition, we asked about Sarcopenia—a nutritional disorder which is frequently overlooked in elderly patients [4, 15, 16].

### Data analysis

Data analysis was conducted using SPSS v. 27.0 (IBM Corp., Armonk, NY, USA). Descriptive statistics were used to depict the respondents’ level of agreement with each of the statements, using the three following categories: (1) ‘agree’ (a combination of *completely agree* and *agree*); (2) ‘partially agree’; and (3) ‘disagree’ (a combination of *completely disagree* and* disagree*). Chi square was calculated to compare responses by gender and years of experience.

## Results

A total of 126 physicians participated in the study (67% females). As seen in Table 1, their mean age was 47.2 years (SD ± 12.6), Most were board certified family physicians (92%) who had studied medicine in Israel (75%). The mean number of total years of experience as a physician was 15.6 years (SD 12.5) and as a FP 10.2 years (SD 11.3).

**Table 1:** Respondents’ demographic characteristics (N = 126)

Characteristic	Data
Age	M(SD) = 47.2 ± 12.6
Females	n = 84 (67%)
Family physicians	n = 112 (92%)
Studied medicine in Israel	n = 94 (75%)
Years of experience as physician	M(SD) = 15.6 ± 12.5
Years of experience as Family physicians	M(SD) = 10.2 ± 11.3

As seen in Table 2, most FP's (92.7%) agreed that diagnosing malnutrition is important. Similarly, most FPs (77.6%) agreed that in elderly patients with decreased appetite, nutritional assessments are necessary. However, only about one-third (35.2%) stated that they have adequate knowledge in the field of nutrition, and less than half (46.4%) stated that nutritional screening should be performed by nurses rather than by physicians. About one-third (35.5%) agreed that involuntary weight loss should be treated with oral nutritional supplement (ONS). Finally, 38.9% of the respondents attributed lack of time and lack of personnel as barriers in screening for malnutrition. No significant differences were found between the respondents by demographics (age, gender, years of experience as FP, place of studying medicine).

**Table 2:** Attitudes of family physicians towards diagnosing and treating malnutrition in the elderly

		Fully agree n (%)	Partially agree n (%)	Disagree n (%)
1	In my opinion, the knowledge I have in the field of nutrition is sufficient to help my elderly patients	33 (26.4)	44 (35.2)	48 (38.4)
2	It's more important to treat obesity than malnutrition	5 (4.0)	11 (8.7)	110 (87.3)
3	It's important to identify malnutrition in elderly patients, to improve their quality of life	117 (92.9)	5 (4.0)	4 (3.2)
4	Screening for malnutrition in the elderly should be performed by nurses, who should refer them to a dietician if needed	58 (46.4)	44 (35.2)	23 (18.4)
5	When patients report a loss of appetite, they should undergo a nutritional assessment	97 (77.6)	20 (16.0)	8 (6.4)
6	When elderly patients report having lost 2–3 kg in three months, the physician should recommend that they consume oral nutritional supplements, such as Ensure or Nutren	12 (9.7)	44 (35.5)	68 (54.8)
7	Carrying out nutritional screening of elderly patients in the clinic is not possible, due to lack of time and personnel	49 (38.9)	37 (29.4)	40 (31.7)

Table 3 describes Family Physician's knowledge on the diagnosis and treatment of malnutrition in the elderly. Almost two-thirds of the respondents (63.5%) stated that they are knowledgeable in nutritional screening principles, and most (83.2%) stated that elderly people who are obese could also suffer from malnutrition. Very few (4%) agreed that low albumin levels are *not* necessarily an indicator of malnutrition [14]. Just over half the respondents (60%) agreed that normal body-mass index (BMI) values in the elderly differ from those in younger populations, and more than half (57.1%) agreed that Sarcopenia should be treated by referring the patient to a dietician. Additionally, almost all respondents (91%) agreed that two specific questions regarding the nutritional status of elderly patients should be an integral part of their medical visits. Moreover, the majority (71%) of physicians agreed to receive training on how to identify malnutrition and how to use a range of screening tools to detect malnutrition. No significant differences were found between the respondents by demographics.

**Table 3:** Knowledge of family physicians regarding diagnosing and treating malnutrition in the elderly

		Fully agree N (%)	Partially agree N (%)	Disagree N (%)
1	Elderly people who are obese cannot suffer from malnutrition as well	7 (5.6)	14 (11.2)	*104 (83.2)
2	When an elderly patient is in a state of sarcopenia (loss of muscle strength and muscle mass), I refer him/her to a dietician	72* (57.1)	36 (28.6)	18 (14.3)
3	Older patients who are overweight should be encouraged to lose weight	42 (33.3)	40 (31.7)	*44 (34.9)
4	Normal BMI values in the elderly (70 years and older) differ from those of younger individuals	76* (60.0)	20 (15.9)	30 (23.8)
5	A lower-than-normal albumin value may indicate a state of malnutrition and requires additional lab tests	103 (81.7)	18 (14.3)	*5 (4.0)
6	It's important to perform nutritional screening once a year for identifying malnutrition in the elderly using a validated screening tool	*80 (63.5)	25 (19.8)	21 (16.7)

*Expected answer

## Discussion

Malnutrition diagnosis and management in primary care is a significant clinical issue as recently highlighted [17, 18]. Our study found that FPs recognize the importance of screening and diagnosing malnutrition in the elderly despite barriers such as lack of time, lack of knowledge, and high workload. In addition, respondents were willing to add two questions annually on screening for malnutrition risk in their elderly patients. Thus, the findings offer insights into how to overcome evidence-practice gaps such as screening, assessment, and diagnosis of malnutrition in primary care as indicated in the recent review [14].

Malnutrition leads to increased healthcare costs, with malnourished individuals requiring more health care professional consultations, hospitalization, health care monitoring and treatments [8] Elderly at risk of malnutrition reported having used more health care services [19]. In addition, malnutrition is associated with impaired quality of life [20]. In a study in Israel, the patients at risk of malnutrition who were admitted to an acute care hospitalization visited their FP more often during the year before hospitalization compared to those hospitalized with no risk of malnutrition [21].

Previous studies focusing on malnutrition in primary care were mostly based on qualitative research methods, which can explain some of the differences in the current study's findings. We found that FPs recognize the importance of diagnosing malnutrition compared to the evidence in the literature that suggests otherwise. In other words, FPs view malnutrition in older adults as a secondary concern compared to other higher priority issues presented by patients [22]. In line with previous studies, FPs did not agree that oral nutritional supplement is the first-line approach to treating malnutrition [23]. The barriers mentioned in this study such as a lack of time, overwhelming workload, lack of awareness and knowledge are consistent with the barriers found in other studies [24]. A Delphi study among Dutch FPs showed that the top disease-related nutrition topics were diabetes mellitus, hypercholesterolemia, and obesity, whereas nutrition problems to older people ranked as the lowest priority [25]. This reflects the historical focus in medical training where nutrition education has been traditionally used for the prevention of cardiovascular diseases [24]. Hence, the findings of the current study, where FPs gave an equal weight to the importance of treating obesity and malnutrition are impressive. Finally, to the best of our knowledge, no literature has been found regarding FPs attitudes and knowledge on interventions for Sarcopenia and appetite change, which are included in this study.

**Fig. 1:**
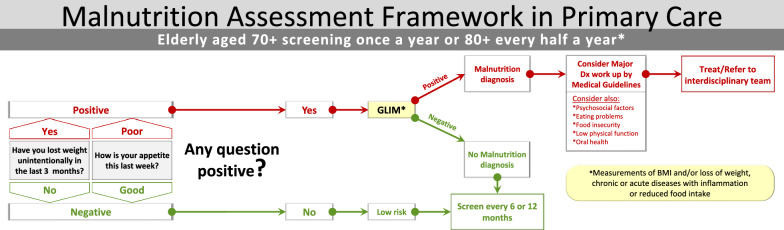
Malnutrition Assessment Framework in Primary Care

We propose an algorithm (Fig. 1) for screening the elderly for the risk of malnutrition using two questions which have been identified as predictors or as signs of malnutrition [4]. The screening will be conducted by either the FPs, other staff members, or self-administered. If the answer to one of the questions is positive, we recommend that the physician will record a diagnosis of malnutrition in the patient’s electronic medical records (using the future ICD-11 classification), [1]. After recognizing malnutrition as a diagnosis, according to the GLIM criteria, the FP will precede with a medical work up for identifying the reasons for malnutrition, and. refer to the interdisciplinary team for treatment.

The GLIM framework states that one of the following phenotypes (weight loss, low BMI, or low-fat free mass), must be positive in conjunction with one of the two etiologies, (reduced food intake/ assimilation or disease with inflammation) in order to classify the patient as malnourished. The data relating to weight loss and BMI are written in the patients' medical records and is also part of the quality indicators which are monitored in community medicine in Israel. The etiology of a disease with inflammation is either diagnosed in the electronic medical files or detected in the physician’s workup. When the patient has a diagnosis of malnutrition by the ICD-11 classification, this diagnosis will be part of the patient’s medical records when he is seen by any other specialist or hospital. This diagnosis will be a basis for triggering a comprehensive interdisciplinary intervention based on the organizations’ policy. As most of the physicians agreed to receive training in how to use a variety of screening tools to detect the risk of malnutrition and diagnose malnutrition, we propose creating a short electronic interactive tutorial relating to the GLIM criteria framework.

The three limitations of the current study are the low response rate of the physicians. As a result, we sent the questionnaire to the same original list via two other recruitment channels. This might have introduced a selection bias into the findings. Another limitation is the higher representation of younger FPs trained in Israeli medical schools and board-certified FP's who might be more oriented/knowledgeable in the subject of malnutrition than the general FP's working in the community. In addition, as all data was electronically sent to one data base for analysis, we cannot distinguish between the three recruitment channels.

Improving the nutritional knowledge of FPs is crucial as they play a significant role in providing primary healthcare and in referring the malnourished elderly to a dietitian or other relevant professions for treatment.

The current study is one of the first studies to use a short survey to provide a basic understanding for developing the proper intervention for FPs. By utilizing a short.

nutritional screening method, nutritional risk can be identified and followed by the GLIM to diagnose malnutrition. Of particular significance is the potential integration of this process into an artificial intelligence [26] framework, to enhance FPs' awareness regarding the nutritional status of their patients, leading to timelier interventions and improved patient outcomes.

## Conclusion

FPs, recognize the importance of screening and diagnosing malnutrition in the elderly despite barriers such as lack of time, lack of knowledge, and high workload. In addition, respondents were willing to add two questions annually on screening for malnutrition risk in their elderly patients. Based on previous publications and our findings we propose (Fig. 1) a simple algorithm for malnutrition diagnosis and intervention using GLIM and existing medical records data. The algorithm begins with screening, identifying patients at nutritional risk followed by the GLIM to diagnose malnutrition and referring malnourished patients to the proper intervention. We believe that the algorithm can be implemented into the medical records as part of preventive measures to improve the health and quality of life of the malnourished elderly patients.

## Data Availability

The data that support the findings of this study are available from the corresponding author on reasonable request.

## Appendix 1. Study Questionnaire

**Table:**

1	In my opinion, the knowledge I have in the field of nutrition is sufficient to help my elderly patients
2	It's more important to treat obesity than malnutrition
3	It's important to identify malnutrition in elderly patients, to improve their quality of life
4	I’m familiar with the guidelines for family physicians regarding the nutritional status of patients
5	Screening for malnutrition in the elderly should be performed by nurses, who should refer them to a dietician if needed
6	When patients report a loss of appetite, they should undergo a nutritional assessment
7	When elderly patients report having lost 2–3 kg in three months, the physician should recommend that they consume oral nutritional supplements, such as Ensure or Nutren
8	Carrying out nutritional screening of elderly patients in the clinic is not possible, due to lack of time and personnel
9	There is a need to evaluate all medications and nutritional supplements taken by elderly patients suffering from poor nutrition
10	Elderly people who are obese cannot suffer from malnutrition as well
11	When an elderly patient is in a state of sarcopenia (loss of muscle strength and muscle mass), I refer him/her to a dietician
12	Older patients who are overweight should be encouraged to lose weight
13	Normal BMI values in the elderly (70 years and older) differ from those of younger individuals
14	A lower-than-normal albumin value may indicate a state of malnutrition and requires additional lab tests
15	It's important to perform nutritional screening once a year for identifying malnutrition in the elderly using a validated screening tool
